# Effect of an automated notification system for deteriorating ward patients on clinical outcomes

**DOI:** 10.1186/s13054-017-1635-z

**Published:** 2017-03-14

**Authors:** Christian P. Subbe, Bernd Duller, Rinaldo Bellomo

**Affiliations:** 1 Respiratory & Critical Care Medicine, Bangor University & Ysbyty Gwynedd, Bangor, LL57 2PW Wales, UK; 2 Clinical Studies & Research Consultant, Perpet Production, Kolumbusstr. 29, 70439 Stuttgart, Germany; 3 Department of Intensive Care, Austin Hospital, 145 Studley Rd, Heidelberg, VIC Australia

## Abstract

**Background:**

Delayed response to clinical deterioration of ward patients is common.

**Methods:**

We performed a prospective before-and-after study in all patients admitted to two clinical ward areas in a district general hospital in the UK. We examined the effect on clinical outcomes of deploying an electronic automated advisory vital signs monitoring and notification system, which relayed abnormal vital signs to a rapid response team (RRT).

**Results:**

We studied 2139 patients before (control) and 2263 after the intervention. During the intervention the number of RRT notifications increased from 405 to 524 (*p* = 0.001) with more notifications triggering fluid therapy, bronchodilators and antibiotics. Moreover, despite an increase in the number of patients with “do not attempt resuscitation” orders (from 99 to 135; *p* = 0.047), mortality decreased from 173 to 147 (*p* = 0.042) patients and cardiac arrests decreased from 14 to 2 events (*p* = 0.002). Finally, the severity of illness in patients admitted to the ICU was reduced (mean Acute Physiology and Chronic Health Evaluation II score: 26 (SD 9) vs. 18 (SD 8)), as was their mortality (from 45% to 24%; *p* = 0.04).

**Conclusions:**

Deployment of an electronic automated advisory vital signs monitoring and notification system to signal clinical deterioration in ward patients was associated with significant improvements in key patient-centered clinical outcomes.

**Trial registration:**

ClinicalTrials.gov, NCT01692847. Registered on 21 September 2012.

**Electronic supplementary material:**

The online version of this article (doi:10.1186/s13054-017-1635-z) contains supplementary material, which is available to authorized users.

## Background

Deterioration of patients on general hospital wards often goes unnoticed for prolonged periods of time [1]. This delay can result in otherwise preventable cardiopulmonary arrest and admission to the intensive care unit (ICU) [2, 3] despite the fact that, in most cases, measurable changes in vital signs [4] could identify patients at risk. Such delayed or absent response to deterioration has been labeled as “failure to rescue” [5], which may be due to a combination of factors [6]. In order to decrease the incidence and consequences of such failure to rescue, many hospitals have introduced rapid response systems (RRSs) [7]. Rapid response systems have been shown to reduce hospital mortality, the rate of cardiopulmonary arrests and preventable ICU admissions, in a number of settings [8]. Nevertheless, even in hospitals with an established RRS, failure-to-rescue events occur [9–11], mostly related to problems with the afferent (monitoring, identification and rapid response team (RRT) activation) component of the RRS. All these failings have in common the dependence on individual bedside staff to raise the alarm.

In contrast to human-based response, industrial high-reliability systems rely on redundancy to ensure that failure of a single part does not result in system failure [12, 13]. When this approach is applied to monitoring in health care, systems with automated notification can be deployed to notify remote and senior healthcare professionals or RRTs who are not at the bedside to respond to deterioration [14, 15]. This approach can be supplemented with continuous monitoring of selected vital signs such as heart rate, respiratory rate and oxygen saturation. Accordingly, we hypothesized that the application of monitoring technology with automated notification of the RRT would improve the reliability of escalation of care for clinically deteriorating patients on general wards, and result in improved patient outcomes.

## Methods

### Setting

We conducted a before-and-after study on two wards of a university-affiliated hospital serving a population of 220,000 inhabitants in the UK. The hospital covers all hospital specialties including a hematology and oncology unit and a dialysis unit but does not provide neuro-surgical, cardiothoracic or transplant surgery care. The ICU has eight beds and there are a further five high dependency and five coronary care beds.

The intervention was undertaken on two general medical wards with 30 and 24 beds, respectively, one admitting patients predominantly with respiratory disease and the other admitting patients with predominantly gastroenterological conditions. The two study wards were selected due to their large proportion of patients with abnormal and complex physiology as evidenced by high rates of critical care transfers and cardiopulmonary arrest prior to the commencement of the study.

### Ethics

The hospital human research ethics committee (Reference 12/WA/0050, Protocol number SD-05163-BBN-IGS A.2) approved the study. Monitoring remained in line with hospital policy in the control and intervention phase. The ethics committee confirmed that no patient consent was required.

### Study patients

We included all patients admitted to the study wards as acute emergencies if they had at least one overnight stay (more than 24 hours of admission). We excluded patients admitted for elective procedures (such as pulmonary biopsy, pleural procedures or endoscopy). Data from all patients were collected on age, gender, main diagnosis (ICD 10 code), date of admission, date of discharge, degree of physiological abnormality as described by the National Early Warning Score (NEWS) [16] on admission, survival status and limitation of medical treatment (LOMT).

### Rapid response system

Hospital policy stipulates the recording of vital signs in acutely unwell patients at least twice per day and with increasing frequency in the presence of increasing severity, usually four times per day. Trained registered nurses and health care assistants obtained and recorded vital signs.

We deployed three different scoring algorithms to identify patients at risk: the NEWS was used as the default algorithm. The Chronic Respiratory Early Warning Score (CREWS) [17] was used in patients with chronic hypoxia due to chronic obstructive pulmonary disease or pulmonary fibrosis. The CREWS algorithm uses different weighting for abnormalities of oxygen saturation. A palliative algorithm scored all patients according to the NEWS algorithm but did not result in notifications. Patients were allocated to either NEWS, CREWS or the palliative algorithm by senior medical staff or the nurse in charge of the ward.

The local vital sign protocol recommends escalation to the nurse in charge of each ward in patients with a score of 3 or more. For a score of 6 or more, it recommends additional escalation to a resident doctor and an advanced nurse practitioner member of the RRT. For a score of 9 or more, it recommends escalation to the lead of the medical on-call team (a physician with a minimum of four years of clinical experience). We obtained data on several key interventions (i.e. fluid bolus, antibiotics, bronchodilators and time on ventilators). In order to avoid subjective judgment, all notifications were classified as independent events.

### Study period

The control and intervention phases were separated by a washout period. Wards were allocated to the intervention phase of the study with a two-month difference. Patient data were collected from 15 October 2012 to 16 October 2013 for the control period in ward 1 and from 1 October 2012 to 2 October 2013 for the control period in ward 2.

Data were collected in the intervention phase from 17 February 2014 in ward 1 and 28 April 2014 in ward 2 until all the interventions ceased in both wards on 17 April 2015. Additional control data were collected in ward 2 from 17 February 2014 to 17 April 2014 to run parallel with the intervention start period in ward 1 for two months.

### Intervention

**Fig. 1:**
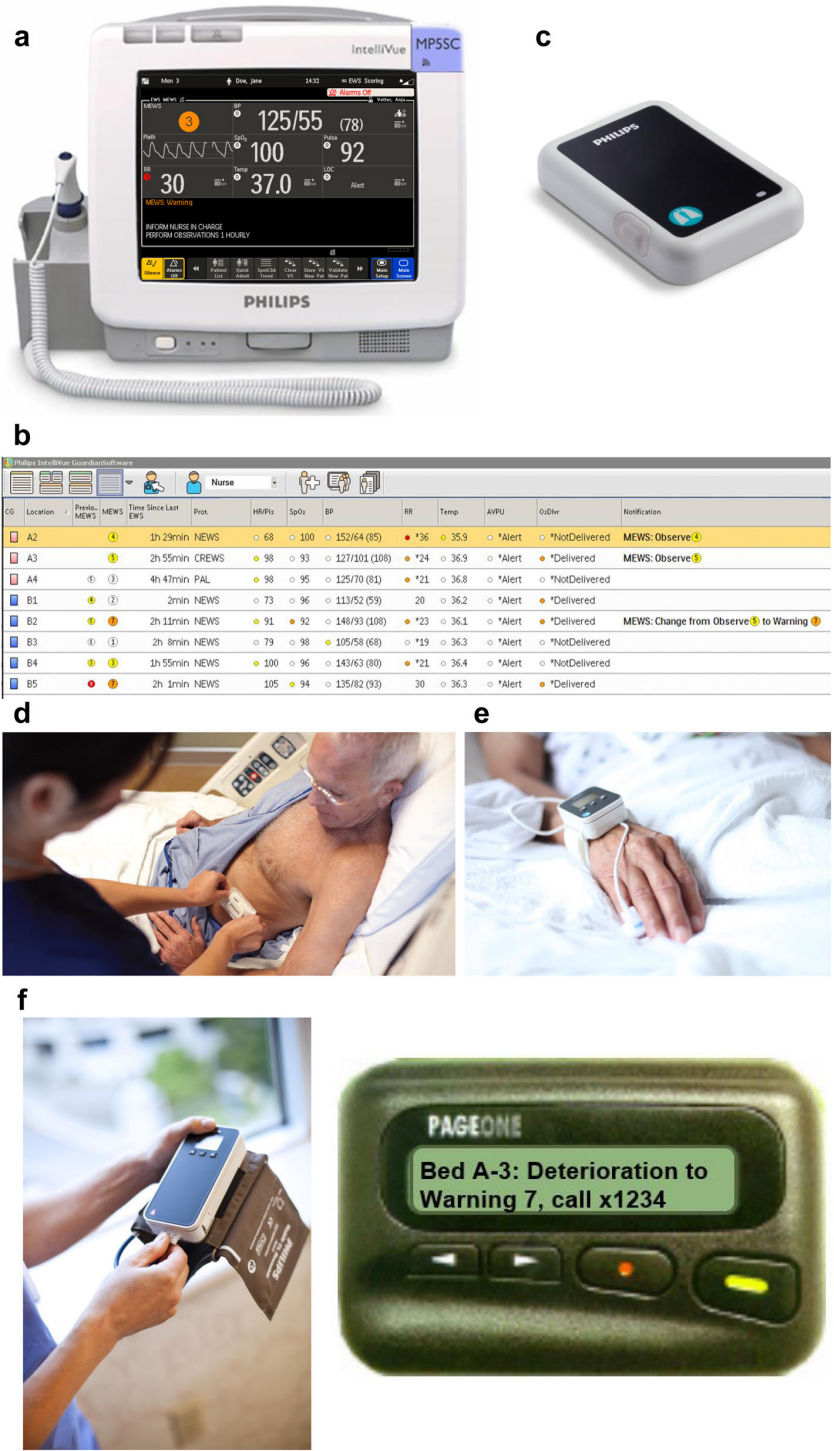
**a**-**f** Elements of the monitoring system with a spot-check monitor with connection to wireless area network (**a**), central screen (**b**), wireless respiratory sensor (**c**), application of wireless respiratory sensor (**d**), wireless oxygen saturation monitor (**e**), wireless blood-pressure monitor (**f**) and alphanumeric paging device (**g**)

During the intervention period an electronic automated advisory vital signs monitoring system (Intelivue Guardian Solution (IGS) with cableless sensors and MP5SC spot-check monitors, Philips Healthcare, Boeblingen, Germany) was deployed to each study ward. The monitoring system electronically transfers and displays respiratory rate, blood pressure, heart rate, pulse oximetry and temperature either obtained by the bedside nurses using spot-check monitors (Fig. 1a) or by cableless sensor devices. The spot-check monitors request the nurse to manually enter respiratory rate (RR) or to confirm an RR measurement from a wireless patient sensor. Information on conscious state was entered manually. Early warning scores (EWSs) were automatically calculated from these vital signs.

The NEWS and CREWS values were displayed and color-coded at central screens (Fig. 1b) in the nursing station as: “safe range” in white; “observe range” in yellow; “warning range” in orange, and “urgent range” in red. The same color codes were used on screens in the offices of consultant physicians of the ward.

Cableless devices for respiratory rate (Philips IntelliVue CL Respiration Pod, Fig. 1c, d) and saturations (Fig. 1e) were set to record vital signs every 15 minutes. Cableless blood pressure devices (Fig. 1f) were set to start a series of 7 measurements every 15 minutes on demand only and at times of EWS. Deployment of cableless sensors was at the discretion of the treating clinicians. Overall, 278 patients had at least one cableless sensor attached during the intervention phase. Based on automated cableless measurements, “deterioration notifications” were paged and were displayed on a big central screen if a change in the cableless parameters resulted in a change of the EWS.

Nurses on the study wards and members of the RRT received notifications about abnormal vital signs via a dedicated alphanumeric paging device (Fig. 1g) that would display the value of the score, the location of the patient and information about the trend (for example “Bed A1, NEWS changed from 5 to 9, call (telephone number)”).

Training in how to use the EWS and the escalation protocol preceded the current study. Training in the use of the IGS was undertaken in two workshops and a dedicated trainer was on site at the hospital for the first week of the intervention period of each of the study wards for in-service training in day and night shifts until 80% of staff had been trained.

### Outcome measures

We obtained information on the prevalence of predefined serious events (acute myocardial infarction, pulmonary embolism, acute pulmonary edema, respiratory failure, stroke, severe sepsis, acute renal failure, emergency admission to the ICU, cardiopulmonary arrest, death), activations of the RRT and patient outcomes, using the hospital administration system and clinical records. Trained research nurses extracted the data.

Data on cardiopulmonary arrests were extracted from clinical notes and cross-referenced with the clinical administration system of the hospital. The same process was followed for deaths in the study wards and admissions to the ICU.

The hospital also collected data as part of the Intensive Care National Audit and Research Centre (ICNARC) case-mix program including data on severity of illness on admission to the ICU as measured by the Acute Physiology and Chronic Health Evaluation II (APACHE II) score [18].

### Statistical analysis

All statistical analysis was performed using Minitab (V16, Minitab Inc., PA, USA) and the Software Package for Social Sciences (SPSS V22). For continuous variables, descriptive statistics are presented as means with standard deviation or 95% confidence intervals (CIs). Where appropriate, medians and interquartile ranges (IQRs) are given. Categorical variables are presented as actual number and proportion of overall count. Continuous variables were compared using the Mann–Whitney test or the Student *t* test as appropriate, while categorical variables were compared using the chi-square, normal approximation or Fisher’s exact test. Binary logistic regression with a backward stepwise (Wald) procedure was used to confirm that mortality and cardiac arrests were independently associated. A *p* value <0.05 was considered statistically significant. The sample size of the study was based on limited data from local spot-check audits: 100 events per phase and ward were considered, according to pertinent nomograms and Gpower software calculations (SIG level 0.05, Power 0.8), to prove a relevant effect.

## Results

### Patient characteristics

During the control period, the 2139 patients with a mean age of 68.8 years (standard deviation (SD) 17 years) were admitted to the study wards compared to 2263 patients with a mean age of 68.6 years (SD 17.3) who were admitted during the intervention period. Patient characteristics are summarized in Table 1. Patients on the respiratory ward had greater severity of illness as measured by NEWS on admission to hospital (2.40, 95% CI 2.24–2.57; *p* < 0.001). The NEWS algorithm was used in 80% of patients, the CREWS algorithm was in used in 16% of patients; 4% of patients were palliative.

**Table 1:** Patient characteristics

	All patients			Patients with RRT notification		
Parameter	Control period	Intervention period	*p*	Control period	Intervention period	*p*
Number of patients	2139	2263		304/2139 (14.2)	365/2263 (16.1)	0.076
Ward 1 (gastroenterology)	941/2139 (44.0)	1062/2263 (46.9)	.051	131/304 (43.1)	177/365 (48.5)	0.163
Ward 2 (pulmonology)	1198/2139 (56.0)	1201/2263 (53.1)	0.051	173/304 (56.9)	188/365 (51.5)	0.163
Age in years	68.8 (68.1–69.5)	68.6 (67.9–69.3)	0.727	71.9 (70.2–73.6)	70.3 (68.7–72.0)	0.200
Male gender	1041/2139 (48.7)	1069/2263 (47.2)	0.343	152/304 (50.0)	175/365 (47.9)	0.596
Ward LOS	7 (4–11)	6 (3–11)	0.232	11 (7–18)	10 (7–19)	0.557
NEWS value on hospital admission	3.27 (3.14–3.41)	3.26 (3.13–3.39)	0.923	3.97 (3.63–4.31)	4.13 (3.81–4.45)	0.511
NEWS value on hospital admission Ward 1	1.84 (1.70–1.98)	2.01 (1.87–2.16)	0.096	2.73 (2.31–3.15)	2.62 (2.24–3.00)	0.692
NEWS value on hospital admission Ward 2	4.30 (4.12–4.48)	4.37 (4.19–4.56)	0.591	4.88 (4.42–5.34)	5.46 (5.04–5.88)	0.068
COPD and CFA diagnosis	526/2139 (24.6)	551/2263 (24.3)	0.851	104/304 (34.2))	122/365 (33.4)	0.831

Data expressed as means with 95% CI, median (IQR) and number (%). *LOS* length of stay, *NEWS* National Early Warning Score, *EWS* early warning score, *COPD* chronic obstructive pulmonary disease, *CFA* cryptogenic fibrosing alveolitis

### Notifications

During the control period, 755/2139 patients (35.3%) reached a score of 6 or more. Their mean age was 72.0 (SD 14.2) years and 387/755 patients (51%) were female. Notification of the RRT, however, occurred only 405 times in 304 patients (1.33 per patient, 189/1000 admissions (18.9%)). The mean age in this patient subgroup was 71.9 (SD 15.1) years. Of these, 320/2139 patients (15.0%) reached a score of 9 or more (Table 2).

**Table 2:** Number of patients with at least one NEWS score above notification limits

Parameter	Control period	Intervention period	*p*	Control period	Intervention period	*p*
NEWS score	6 or more			9 or more		
Number of patients	755/2139 (35.3%)	747/2263 (33.0%)	0.262	320/2139 (15.0%)	302/2263 (13.3%)	0.182
Ward 1 (gastroenterology)	136/941 (14.5%)	135/1062 (12.7%)	0.321	49/941 (5.2%)	49/1062 (4.6%)	0.559
Ward 2 (pulmonology)	619/1198 (51.7%)	612/1201 (51.0%)	0.843	271/1198 (22.6%)	253/1201 (21.1%)	0.460

Vital signs for patients monitored with the Chronic Respiratory Early Warning Score were recalculated using the National Early Warning Score (NEWS) in order to allow for comparison. Data expressed as number (%).

During the intervention period, 747/2263 patients (33.0%) reached a score of 6 or more. Their mean age was 72.3 (SD 14.4) years and 396/747 (53%) patients were female. Notification of the RRT occurred 524 times in 365 patients in the intervention phase (1.43 per patient, 231/1000 admissions (23.1%), *p* = 0.001 for comparison with control period). The mean age of this patient subgroup was 70.3 (SD 15.7). Of these patients, 302/2263 (13.3%) reached a score of 9 or more.

### Response to notifications

During the 929 notifications (405 notifications in 304 patients in the control phase and 524 in 365 patients in the intervention phase), the RRT undertook a range of medical interventions (Table 3) with mortality in affected patients changing from 67/304 (22.0%) in the control phase to 63/365 (17.3%) in the intervention phase (*p* = 0.122).

**Table 3:** Characteristics of patients with notifications and escalation events

Parameter	Control period	Intervention period	*p*
Number of patients	304/2139	365/2263	0.077
Number of notifications	405/2139	524/2263	0.001
Age in years	71.9 (70.2–73.6)	70.3 (68.7–72.0)	0.200
Male gender	152/304 (50.0)	175/365 (47.9)	0.597
EWS value at time of first notification of rapid response team	5.68 (5.23–6.12)	5.53 (5.14–5.93)	0.641
Emergency ICU admissions	26/304 (8.6)	21/365 (5.8)	0.158
Patients with a DNAR order in place	99/304 (32.6)	135/365 (37.0)	0.233
Patients with DNAR order before first notification	35/99 (35.4)	70/135 (51.9)	0.012
Patients with a DNAR order after first notification	63/99 (63.6)	60/135 (44.4)	0.004
Patients with a DNAR order and unknown time	3/99 (3.0)	5/135 (3.7)	0.779
Fluid bolus	193/405 (47.7)	279/524 (53.2)	0.091
Antibiotics	192/405 (47.4)	317/524 (60.5)	<0.001
Bronchodilators	93/405 (23.0)	174/524 (33.2)	0.001
Ventilation in the ICU after RRT review (total hours)	1554.5	752	0.256

Data expressed as means with 95% CI and number (%) unless not indicated otherwise. *DNAR* do not attempt resuscitation, *RRT* rapid response team, *EWS* early warning score

### Outcomes

There were 14 cardiac arrests (6.5/1000 discharges, 3.5% of RRT notifications) in the control period compared with two (0.8/1000 discharges, 0.4% of RRT notifications) in the intervention period (*p* = 0.002). We observed 320 deaths in 4402 patients (mortality 7.3%). Hospital mortality was 173/2139 patients (8.1%) during the control and 147/2263 (6.5%) during the intervention period (difference 1.59%, 95% CI 0.05–3.13%; *p* = 0.042). After exclusion of readmissions, the mortality benefit remained significant in the higher acuity (as measured by higher NEWS) respiratory ward (decrease from 110/983 (11.2%) to 79/945 (8.4%); difference 2.83%, 95% CI 0.19–5.48%; *p* = 0.036), but not in the gastroenterology ward.

Data from the 47 patients admitted to the ICU were extracted from the ICNARC database (1% of 4402 patients) these comprised 26 patients (12/1000 discharges) admitted during the control phase and 21 patients (9/1000 discharges) during the intervention phase (*p* = 0.158). Mean APACHE II scores on admission to the ICU were significantly lower during the intervention phase (18 ± 8 vs. 26 ± 9, *p* < 0.007). This was associated with lower predicted mortality (24% vs. 45%, *p* < 0.006) and a decrease in actual mortality (3/21 (14%) vs. 11/26 (42%), *p* < 0.04).

In total, there were 268 serious events (268 in 2139 patients, 125 per 1000 patients) in the control and 185 serious events (185 in 2263 patients, 82 per 1000 patients) in the intervention period (*p* < 0.001) (Table 4).

**Table 4:** Serious events in the control and intervention period

Parameter	Control period (n = 2139)	Intervention period (n = 2263)	*p*
Number of patients with serious events	208 (9.72)	166 (7.34)	0.005
Total number of serious events	268 (12.53)	185 (8.17)	<0.001
Acute myocardial infarction	4 (0.19)	0	0.056
Pulmonary embolism	3 (0.14)	2 (0.09)	0.679
Acute pulmonary edema	5 (0.23)	1 (0.04)	0.115
Respiratory failure	19 (0.89)	10 (0.44)	0.070
Stroke	0	0	1.000
Severe sepsis	21 (0.98)	1 (0.04)	<0.001
Acute renal failure	3 (0.14)	1 (0.04)	0.361
Emergency admission to the ICU	26 (1.22)	21 (0.93)	0.355
Cardiopulmonary arrest	14 (0.66)	2 (0.09)	0.002
Death	173 (8.09)	147 (6.50)	0.042

Serious events occurred after admission to the study ward and were not the reason for admission. Data expressed as number (%). Denominator: all patients admitted to the study wards (2139 patients in control period, 2263 patients in intervention period). Fisher’s exact test was used instead of the normal approximation test if the number of events in either sample was fewer than five

### Binary logistic regression

Reduced mortality was maintained in stepwise binary logistic regression analysis including age, gender and acuity (measured by type of ward) at step 1: there was reduced mortality for patients admitted during the intervention period (OR = 0.79, 95% CI 0.63–0.99; *p* = 0.043). The same was true for the rate of patients with cardiopulmonary arrest (OR = 0.15, 95% CI 0.03–0.64; *p* = 0.011) (Additional file 1: Table S1).

## Discussion

### Key findings

We conducted a before-and-after study of an electronic automated advisory vital signs monitoring and notification system, which relayed abnormal vital signs to an RRT. We found that implementation of such a system led to a significant increase in RRT notifications and more notifications, which triggered interventions such fluid boluses, bronchodilators, and antibiotics. Moreover, implementation was associated with a significant decrease in cardiac arrests, overall mortality in the sicker of the two clinical areas, the severity of illness in those patients admitted to ICU, and decreased ventilation time and mortality in such patients, and more patients received “do not attempt resuscitation” orders.

### Relationship to previous studies

Automated assessment of physiological abnormalities has been shown to reduce mortality among patients seen by the RRT [19] but the effect on cardiac arrests has not been reported. However, the intervention reduced the number of abnormal sets of vital signs from four sets to three before a call to the RRT was undertaken, implying earlier activation. Introduction of electronic documentation of vital signs was also associated with a reduction in standardized hospital mortality in two large UK university hospitals [20], but it was unclear in which patient group this reduction occurred and whether other concurrent changes might have accounted for the improvement in outcomes.

There is a lack of studies measuring the impact of new monitoring technology on clinical outcomes, with much of the published work focused around technical feasibility [21] and user acceptance [22]. In contrast, we used a combination of wireless sensors to support care in the more unstable patients, in effect facilitating more frequent recording of vital signs with limited need for extra staffing.

### Strengths and limitations

Our study has several strengths. It is the first detailed investigation of the effect of automated notifications on team behavior and clinical outcomes in clinically deteriorating patients. To our best knowledge, this is also the first study of cableless sensors deployed in a clinical trial. The large number of patients, large number of activations, changes in clinical interventions and changes in clinical outcomes documents for the first time that the impact of an automated system across the whole pathway of deterioration in patients was coherent.

Concerns have been raised about the feasibility of implementation of the EWS in patients with respiratory illness [23, 24]. We have shown that by adapting the scoring algorithm, clinically meaningful results can be achieved, even in this group of patients. To our knowledge, this study is also the first that has used a range of scoring algorithms in order to individualize monitoring plans, albeit in a single center.

Our study has some limitations: We did not collect data on admission diagnosis and comorbidity beyond the presence of significant lung disease. It also remains untested whether findings would be different, for example, in a surgical population in whom the number of notifications might be fewer [25, 26] and the patterns of expected physiological derangement likely to be dominated by hypotension.

The fact that RRT responders received automated notification during the intervention period meant that they might have initiated communication more frequently than in the observation period. Resulting phone calls and conversations within parent teams are likely not to have been fully documented in the clinical notes. In the absence of an electronic patient record, we were also unable to find reliable documentation to confirm the timing of interventions. Our study was conducted in a very specific environment and in a UK hospital. Its external validity to other institutions and patient cohorts, therefore, needs to be assessed. Nursing teams were appreciative of the technology used. We are aware that especially with regards to continuous monitoring these finding might not be generalizable [27, 28].

We do not have detailed data to explain the increase in DNAR orders, not only overall, but also before RRT notification. It is possible that the increase is notification might have increased overall awareness of the overall status of all patients and of the limitations of what medical intervention could do for such patients, thereby increasing pro-active decision making on end-of-life care.

Mortality was reduced in the study ward with the sicker patient population. The fact that overall mortality was not affected could be due to the fact that many of the included patients would have suffered from advanced chronic diseases with limited reversibility after a study period of more than two years. Further research is needed in order to define the characteristics of patients who most benefit from the type of intervention described. Finally, the low rates of adverse events limit any clinical inference made from statistical tests related to their incidence during the two study periods.

### Implications of study findings

This study implies that deployment of an electronic automated advisory vital signs monitoring and notification system, which relays abnormal vital signs to an RRT, has the ability to significantly increase the number of activations of the RRT. Moreover, it implies that such an increase in activations can lead to decreased overall mortality, cardiac arrests and illness severity in those patients admitted to ICU, resulting in a specific decrease in mortality among such patients. Finally, our study implies that the more frequent activation affects decision-making on end-of-life care toward greater use of DNAR orders.

### Future research

The findings of our study will need to be confirmed in other patient groups (i.e. surgical, obstetric and pediatric patients) and in other health care settings to tease out how much our findings are context dependent [29]. The mechanisms that result in improved outcomes also require further research: Human-factor-led design with reduction of cognitive load due to a graphic interface or system design with modular redundancy and increase in the number of staff members to are aware of emerging deteriorations might be candidate mechanisms.

## Conclusions

Implementation of an electronic automated advisory vital signs monitoring and notification system appears to increase RRT notifications and RRT interventions associated with decreases in cardiac arrests, overall mortality, illness severity and mortality in those patients admitted to ICU and an increase in pro-active decision-making on end-of-life care. Wider testing of this system in different populations and health care settings now appears justified.

## Additional file

Additional file 1: Table S1.
**a** Results of the multivariate stepwise binary logistic regression for mortality (backward Wald). **b** Results of the multivariate stepwise binary logistic regression for cardiac arrest patients (backward Wald). (DOCX 22 kb)
