# Supplementary material for: Effect of an automated notification system for deteriorating ward patients on clinical outcomes
Source: Crit Care. 2017 Mar 14;21:52. doi: 10.1186/s13054-017-1635-z (PMC5348741; doi:10.1186/s13054-017-1635-z)
Supplement: Additional file 1: Table S1. — a Results of the multivariate stepwise binary logistic regression for mortality (backward Wald). b Results of the multivariate stepwise binary logistic regression for cardiac arrest patients (backward Wald). (DOCX 22 kb) [file 13054_2017_1635_MOESM1_ESM.docx]

| **Table S1a:** Results of the multivariate stepwise binary logistic regression for mortality (Backward Wald) | | | | | | | | | |
| --- | --- | --- | --- | --- | --- | --- | --- | --- | --- |
|  | | B | S.E. | Wald | df | p | OR | 95% CI for OR | |
|  |  |  |  |  |  |  |  | Lower | Upper |
| Step 1^a^ | Age | .036 | .004 | 69.651 | 1 | .000 | 1.037 | 1.028 | 1.046 |
|  | Acuity | .119 | .119 | .990 | 1 | .320 | 1.126 | .891 | 1.423 |
|  | Intervention | -.232 | .118 | 3.889 | 1 | .049 | .793 | .630 | .999 |
|  | Gender | .049 | .118 | .175 | 1 | .676 | 1.051 | .834 | 1.324 |
|  | Constant | -5.168 | .356 | 210.209 | 1 | .000 | .006 |  |  |
| Step 2 | Age | .036 | .004 | 69.843 | 1 | .000 | 1.037 | 1.028 | 1.046 |
|  | Acuity | .120 | .119 | 1.010 | 1 | .315 | 1.127 | .892 | 1.424 |
|  | Intervention | -.233 | .118 | 3.907 | 1 | .048 | .793 | .629 | .998 |
|  | Constant | -5.132 | .345 | 220.982 | 1 | .000 | .006 |  |  |
| Step 3 | Age^b^ | .036 | .004 | 70.893 | 1 | .000 | 1.037 | 1.028 | 1.046 |
|  | Intervention^b^ | -.238 | .118 | 4.083 | 1 | .043 | .789 | .626 | .993 |
|  | Constant | -5.069 | .338 | 225.333 | 1 | .000 | .006 |  |  |
| B, parameter estimate; OR, odds ratio; CI, confidence interval; p < .05 was considered significant; ^a^Variables entered on step 1: Age, Acuity, Intervention, Gender; ^b^remaining variables after stepwise backward elimination (Wald); Hosmer-Lemeshow (step 3) chi-square 9.952, df = 8, p = .268; Nagelkerke R^2^ = .050. | | | | | | | | | |

| **Table S1b:** Results of the multivariate stepwise binary logistic regression for cardiac arrest patients (Backward Wald) | | | | | | | | | |
| --- | --- | --- | --- | --- | --- | --- | --- | --- | --- |
|  | | B | S.E. | Wald | df | p | OR | 95% CI for OR | |
|  |  |  |  |  |  |  |  | Lower | Upper |
| Step 1^a^ | Age | .019 | .017 | 1.272 | 1 | .259 | 1.019 | .986 | 1.054 |
|  | Acuity | -.436 | .522 | .698 | 1 | .404 | .647 | .232 | 1.799 |
|  | Intervention | -1.949 | .761 | 6.565 | 1 | .010 | .142 | .032 | .633 |
|  | Gender | .263 | .523 | .253 | 1 | .615 | 1.301 | .467 | 3.624 |
|  | Constant | -6.373 | 1.338 | 22.704 | 1 | .000 | .002 |  |  |
| Step 2 | Age | .018 | .017 | 1.185 | 1 | .276 | 1.018 | .985 | 1.053 |
|  | Acuity | -.423 | .521 | .659 | 1 | .417 | .655 | .236 | 1.819 |
|  | Intervention | -1.948 | .761 | 6.563 | 1 | .010 | .143 | .032 | .633 |
|  | Constant | -6.179 | 1.268 | 23.757 | 1 | .000 | .002 |  |  |
| Step 3 | Age | .017 | .017 | 1.026 | 1 | .311 | 1.017 | .984 | 1.052 |
|  | Intervention | -1.932 | .760 | 6.460 | 1 | .011 | .145 | .033 | .643 |
|  | Constant | -6.320 | 1.275 | 24.568 | 1 | .000 | .002 |  |  |
| Step 4 | Intervention^b^ | -1.934 | .760 | 6.472 | 1 | .011 | .145 | .033 | .641 |
|  | Constant | -5.097 | .278 | 335.623 | 1 | .000 | .006 |  |  |
| B, parameter estimate; OR, odds ratio; CI, confidence interval; p < .05 was considered significant; ^a^Variables entered on step 1: Age, Acuity, Intervention, Gender; ^b^remaining variables after stepwise backward elimination (Wald); Hosmer-Lemeshow (prior to step 4) chi-square 3.112, df = 8, p= .927; Nagelkerke R^2^ = .060. | | | | | | | | | |
